# Breast tubular adenoma: diagnostic challenges and management insights from a six-case series

**DOI:** 10.1093/jscr/rjag865

**Published:** 2026-09-29

**Authors:** Wei Ren Ng, Samantha Pellegrino, Clara Macindoe, Maryam Shamassi, Hassan Shoushtari Zadeh, Henry To

**Affiliations:** Department of General Surgery, Werribee Mercy Hospital, 300 Princes Highway, Werribee, Victoria 3030, Australia; Department of General Surgery, Werribee Mercy Hospital, 300 Princes Highway, Werribee, Victoria 3030, Australia; Anatomical Pathology, Dorevitch Pathology, 18 Banksia Street, Heidelberg, Victoria 3084, Australia; Anatomical Pathology, Dorevitch Pathology, 18 Banksia Street, Heidelberg, Victoria 3084, Australia; LUMUS Imaging, Werribee Mercy Hospital, 300 Princes Highway, Werribee, Victoria 3030, Australia; Department of General Surgery, Werribee Mercy Hospital, 300 Princes Highway, Werribee, Victoria 3030, Australia; University of Melbourne, Grattan Street, Parkville, Victoria 3010, Australia

**Keywords:** tubular adenoma, benign breast tumour, breast mass, histopathology, surgical excision, surveillance

## Abstract

Tubular adenoma is a rare benign breast tumour that is often indistinguishable from fibroadenoma on clinical examination and imaging, making diagnosis and management challenging. We present a six-patient case series describing the clinical, radiological, and histopathological features of this uncommon lesion. Patients ranged from 22 to 62 years of age. Ultrasound consistently demonstrated well-circumscribed hypoechoic lesions with variable internal vascularity, while core biopsy established the diagnosis in all cases. Four patients underwent surgical excision, confirming benign tubular adenoma, and two were managed with surveillance following radiological–pathological concordance. No recurrence, interval growth, or malignant transformation was observed during follow-up. Although tubular adenoma is benign, rare reports of synchronous or adjacent breast carcinoma highlight the importance of histopathological confirmation and careful follow-up. Our findings suggest that selective surveillance may be considered in patients with radiological–pathological concordance, while surgical excision remains appropriate in cases of diagnostic uncertainty, lesion progression, or radiological–pathological discordance.

## Introduction

Tubular adenoma is a rare benign epithelial breast tumour that predominantly affects women of reproductive age and typically presents as a painless, mobile, well-circumscribed breast mass that is clinically and radiologically indistinguishable from the more common fibroadenoma. Consequently, definitive diagnosis relies on histopathological examination. Although tubular adenoma is considered a benign lesion with an excellent prognosis, rare reports of synchronous or adjacent breast carcinoma have raised uncertainty regarding its optimal management. Current evidence is limited to isolated case reports and small case series, and there are no established evidence-based management guidelines. We present a six-patient case series describing the clinical presentation, radiological and histopathological features, management strategies, and follow-up outcomes of breast tubular adenoma. Our findings contribute to the limited literature supporting histopathological confirmation and selective surveillance in patients with radiological–pathological concordance, while reinforcing the role of surgical excision when diagnostic uncertainty or discordance exists.

## Case series

Case A is a 40-year-old female presenting with an incidental right breast lesion following a minor, ipsilateral self-limiting non-lactational mastitis. She has a 23-year history of oral contraceptive pill use, and her mother was diagnosed with breast cancer at the age of 59. There were no other risk factors of concern, and she was otherwise well. On examination there was a diminutive palpable lesion at the peri-areolar 3 o’clock position, and no other breast lesion. Breast ultrasound described a 7 mm hypoechoic, homogenous, and well-defined lesion with mild internal vascularity (BI-RADS 2). There were no other breast lesions. The core biopsy showed an epithelial-predominant adenosis-like nodular fibro-epithelial lesion, in keeping with a tubular adenoma. The patient opted to have the lesion monitored. Two repeat ultrasounds over the next 18 months did not reveal any change in the size or characteristics of the lesion or any new breast lesions.

Case B is a 34-year-old female who had a 6-month history of bilateral palpable breast lumps. She has a history of ulcerative colitis and prior ovarian cyst removals but no history of breast disease. Her mother was diagnosed with breast cancer after the age of 50. She was not taking the oral contraceptive and has one child aged 7 years old. On examination, there were two palpable breast lumps, 6 o’clock on the right and 11 o’clock on the left, both of which were mobile and non-tender (Fig. 1).

**Figure 1:**
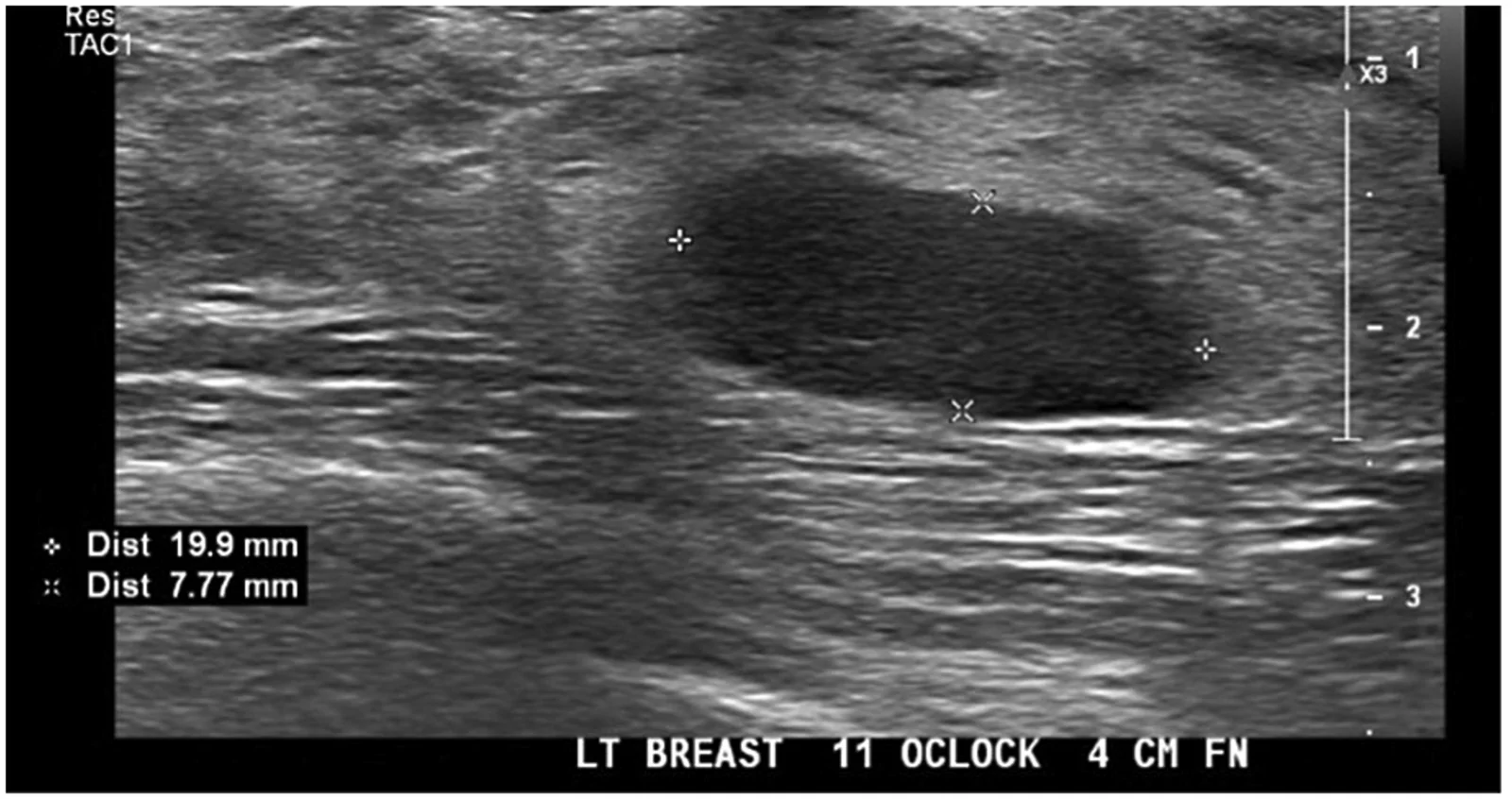
Well-defined, ovoid, and hypoechoic lesion with posterior enhancement and distinct border with no internal vascularity.

Bilateral ultrasound demonstrated an 18 mm right breast lesion at 6 o’clock with some internal vascularity and a 20 mm left breast lesion at 11 o’clock with no internal vascularity. Both were well-defined hypoechoic lesions (BI-RADS 2). However, core biopsies showed that the right breast lesion was a fibroadenoma, while the left breast lesion was a tubular adenoma with typical tubuloglandular structures lined by bland myoepithelium. As the patient was pregnant at the time, both lesions were monitored with ultrasonography over the next 12 months and remained stable. The patient opted for no further treatment.

Case C is a 62-year-old post-menopausal female who was referred for a new palpable left breast lump at the 6 o’clock position. Her mother was diagnosed with breast cancer in her sixties, along with two other members of her family, including a great aunt and second cousin. She has three adult children, and no hormonal exposures.

A left breast ultrasound found an indeterminate 6 o’clock heterogenous, lobulated 14 mm lesion with some internal vascularity (BI-RADS 2), not seen on previous imaging 2 years prior. There was also a new incidental ovoid, well-circumscribed lesion at 2 o’clock, measuring 8 mm (BI-RADS 2). A core biopsy targeting these two new lesions found the 6 o’clock lesion to be a tubular adenoma with multiple crowded tubular structures with minimal surrounding fibrous stroma. The 2 o’clock lesion was a fibroadenoma.

Given her family history of breast cancer, an excisional biopsy was performed. The histopathology showed a completely excised 11 mm lesion with crowded uniform tubular structures and mildly myxoid stroma with no malignant features, consistent with a tubular adenoma. Ultrasounds at 6 and 12 months post-excision did not reveal any new abnormalities (Fig. 2).

**Figure 2:**
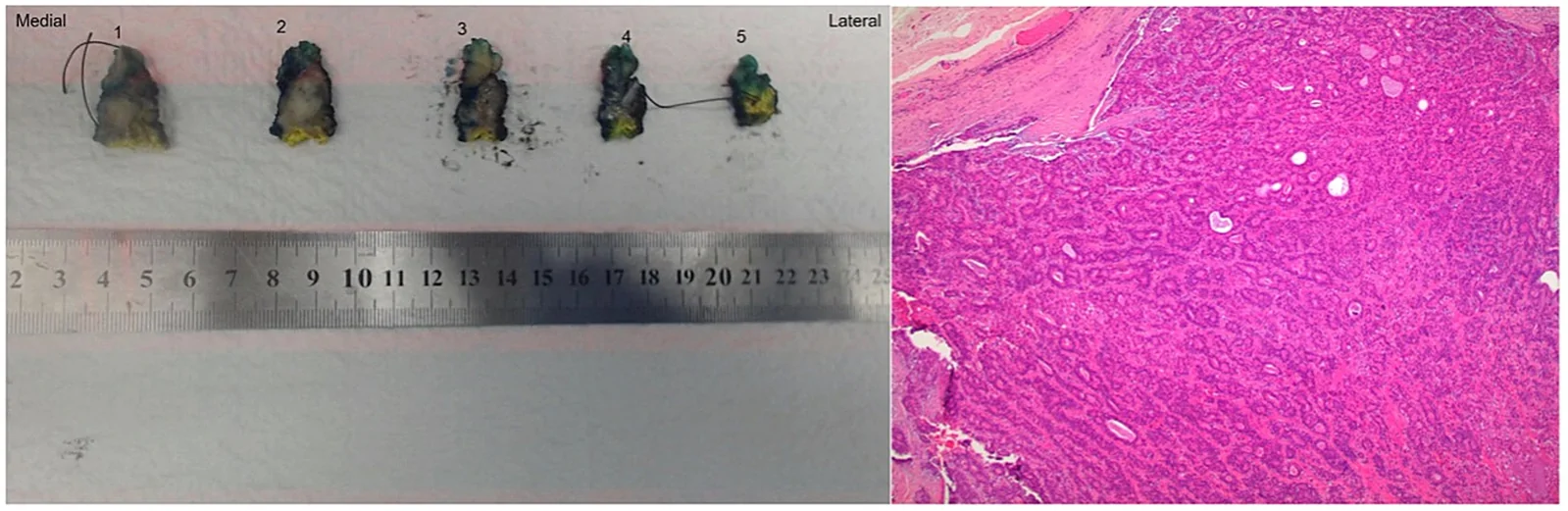
Macroscopic examination demonstrated a well-circumscribed 11 × 11 × 9 mm pale tan breast lesion. Microscopy showed uniform tubular structures with dual epithelial and myoepithelial cell layers within mildly myxoid stroma, consistent with completely excised tubular adenoma without malignancy.

Case D is a 30-year-old female who presented with a 2-year history of a left breast lump at 1 o’clock with cyclical mastalgia. Previous imaging showed variable sizing of the lesion of interest, with initial size at 18 mm, subsequently 13 and 16 mm on surveillance imaging. The lesion was described as a well-defined, hypoechoic lesion with no internal vascularity (BI-RADS 2). She has no risk factors for breast cancer and no known family history of breast cancer. Given the prolonged nature of her symptoms, she opted to have a core biopsy and subsequent excision biopsy.

Core biopsy revealed benign fibroadipose breast parenchyma with features of tubular adenoma. Excisional biopsy confirmed a circumscribed fibroepithelial lesion lined by a dual cell layer of epithelial and myoepithelial cells with surrounding collagenous stroma consistent with a tubular adenoma. Surveillance imaging at 5 months post-surgery showed no evidence of recurrence (Fig. 3).

**Figure 3:**
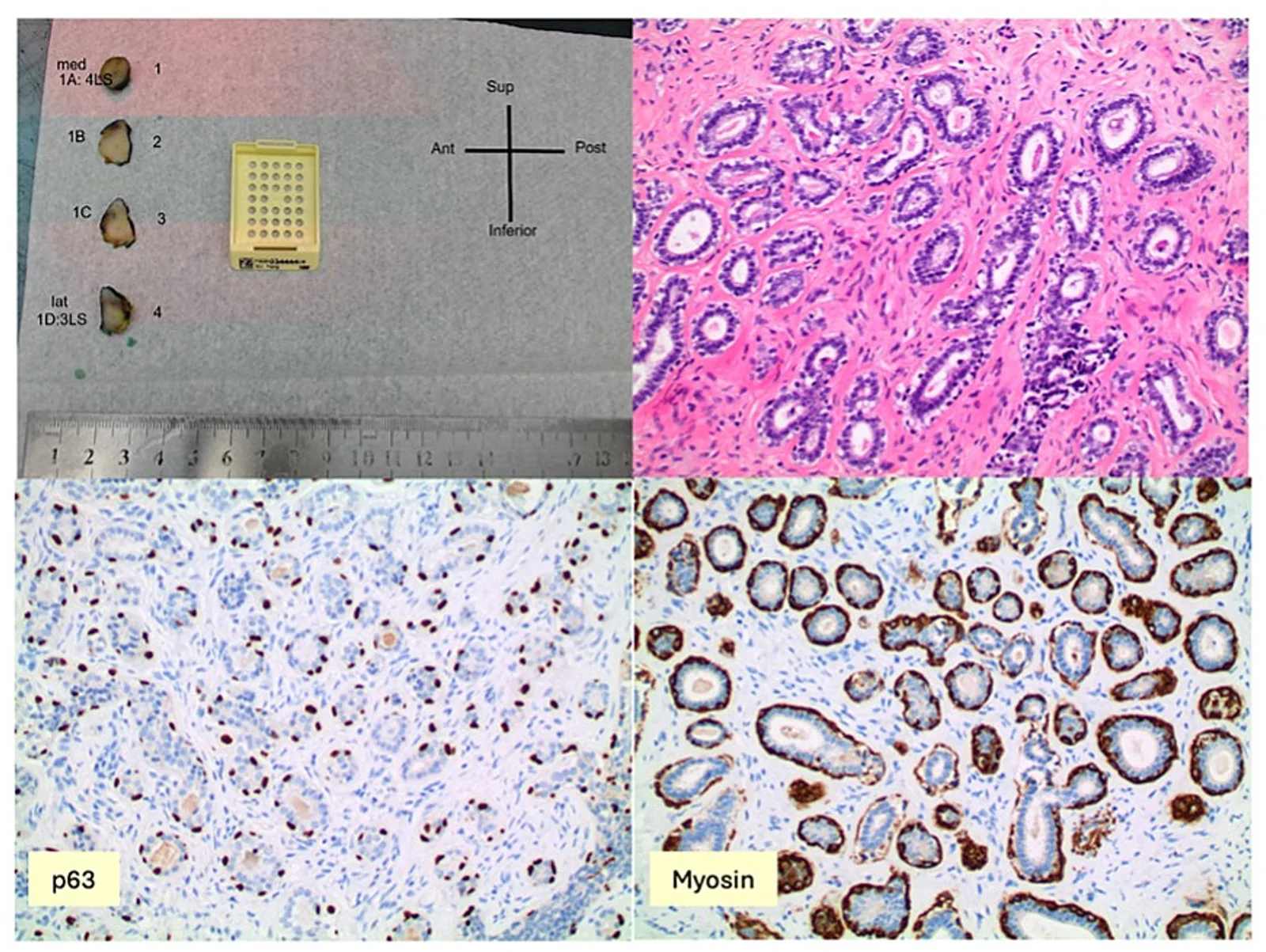
Macroscopic examination demonstrated a 16 × 14 × 10 mm white firm breast lesion. Microscopy showed a circumscribed fibroepithelial proliferation with branching ducts, dual epithelial–myoepithelial cell layers, and collagenous stroma, consistent with benign tubular adenoma without malignancy.

Case E is a 22-year-old female who was referred with a 13 mm lobulated, hypoechoic lesion in the right breast at 3 o’clock. It was well-defined and had no internal vascularity (BI-RADS 2), and was palpable on examination. She has a positive family history of breast cancer in her maternal grandmother in her sixties and had oral contraceptive use for 5 years. Core biopsy showed adenosis-like proliferation of open and branching ductules lined by a dual layer of uniform epithelial and myoepithelial cells consistent with tubular adenoma (Fig. 4).

**Figure 4:**
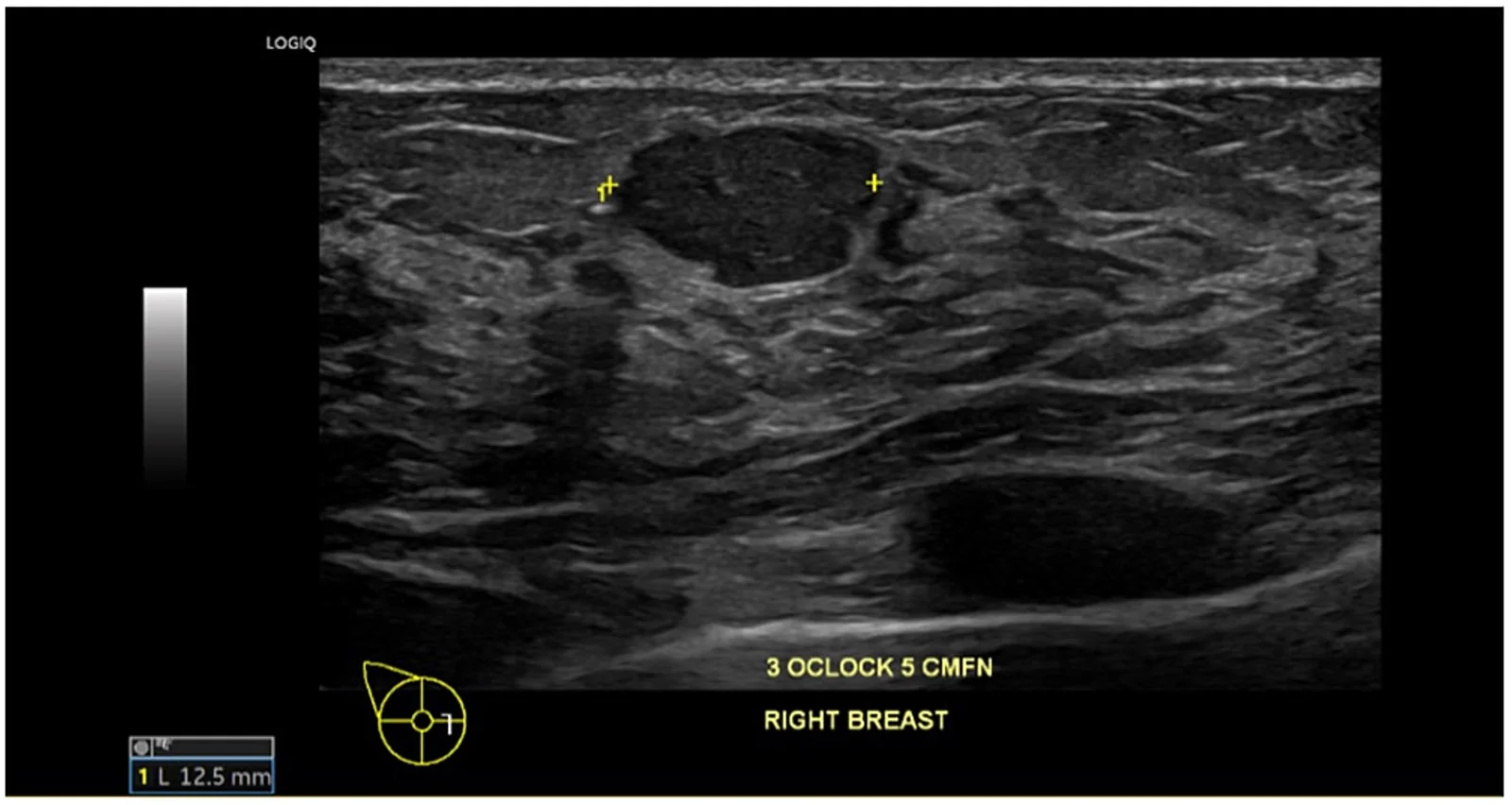
Well-defined, ovoid, and lobulated hypoechoic lesion with posterior enhancement and distinct border with no internal vascularity.

Following discussion with the patient, the decision was made for an excisional biopsy, which confirmed a completely excised tubular adenoma with adenosis-like proliferation of focally branching ductules lined by dual cell layers. Surveillance imaging 7 months post-surgery showed residual scar tissue in the area of interest.

Case F is a 36-year-old pregnant female referred with a palpable right breast lump first noted 4 years prior. A previous biopsy 4 years ago was reported as benign. However, she noted an increase in size of the lump in the past 6 months during her pregnancy. Her paternal aunt had breast cancer in her fifties, but she has no significant past medical history. Physical examination revealed a single mobile, non-tender lesion in the right axillary tail with no overlying skin changes. Ultrasound showed a 51-mm well-defined, circumscribed hypoechoic lesion with linear echogenic septi with some internal vascularity (BI-RADS 4). A core biopsy showed a lesion composed of benign epithelial and stromal elements and epithelium formed with closely packed tubules, consistent with tubular adenoma.

As she was pregnant at the time of diagnosis, a decision was made for an excisional biopsy after delivery. Given her breastfeeding status at the time of surgery, the complete excisional biopsy showed hyperplastic lobules with lactational changes consistent with a benign hamartoma. While the pre-operative diagnosis with the core biopsy showed tubular adenoma, the lactational changes present at the time of surgery likely distorted the overall appearance such that the tubular adenomatous changes were less appreciable. Following multi-disciplinary discussion, the initial diagnosis of tubular adenoma still stands, and it was completely excised. She will undergo follow-up surveillance ultrasound in 12 months to ensure that there is no recurrence.

## Discussion

Our case series of six patients highlights a histopathological diagnosis of breast tubular adenoma, a rare benign lesion with an incidence ranging from 0.13% to 2.9%. These typically occur in premenopausal women of childbearing age with ~ 90% of cases occurring in women under 40 years of age [1, 2]. They are thought to be very rare after menopause and are not commonly associated with oral contraceptive use or pregnancy [3].

The aetiology of tubular adenomas is not completely known but is thought to have a correlation with reproductive hormones due to the higher incidence in premenopausal women. This phenomenon was also seen in male patients with cirrhosis, where Clark et al. described a possible link between elevated oestrogen in cirrhotic patients and breast tubular adenoma [4]. This also coincided with five of our cases, but Case C presented with this lesion 10 years after menopause. While less common in postmenopausal women, there has been a report of an 84-year-old with tubular adenoma [5]. The typical location for tubular adenomas is in the upper-outer quadrant of the breast [6].

There has also been evidence to suggest a common histogenetic mechanism between breast tubular adenomas and fibroadenomas, given that the two histopathologic findings have been found in the same specimen in some case reports [7]. In our series, Case B had both a fibroadenoma and tubular adenoma, but from separate lesions taken from opposite breasts.

Improving presurgical diagnostic confidence with both accepted radiological and pathological features would be valuable in cases where nonsurgical management is opted, and a more informed and confident discussion between patient and clinician can occur, giving the patient more reassurance about the benign nature of the diagnosis.

Fibroadenomas typically have an abundant stromal component compared to tubular adenoma, while tubular adenoma has tightly packed tubular and acinar epithelial components, with uniformly sized ducts lined by epithelium and myoepithelium [8]. However, significant overlap of these features can further complicate an accurate diagnosis [9]. One recent study reported a statistically significant difference in histology between tubular adenoma and fibroadenoma. Tissue fragments with multiple tubular structures are more commonly seen in tubular adenoma, and are devoid of malignant features, while larger staghorn or honeycomb epithelial sheets are seen in fibroadenomas [10].

On clinical examination, these lesions are freely mobile, well-defined and usually painless. There are usually no overlying skin changes or nipple alteration, and they clinically resemble fibroadenomas, a much more common benign breast tumour [8]. Typical ultrasound imaging would show a well-circumscribed hypoechoic lesion; however, our cases have shown variability in this area [6]. As such, a definitive diagnosis of tubular adenoma can only be satisfied with a histopathological examination.

An important point is recognizing that the histologic differential diagnoses of breast tubular adenomas range from benign to malignant tumours, hence the critical significance in accurately identifying this rare histopathologic finding. Rare instances of ductal carcinoma *in situ* arising within tubular adenomas have been reported [9]. Another such case report showed findings of tubular adenoma and invasive ductal carcinoma in the same specimen. While the case reported clearly defined histological boundaries between tubular adenoma and invasive ductal carcinoma, a malignant transformation cannot be ruled out [11]. Fine-needle aspiration alone is insufficient for a definitive diagnosis and often necessitates complete surgical excision of the lesion [6].

In the event of radiological–pathological discordance, the American Society of Breast Surgeons recommends further histological evaluation, either in the form of a repeat core biopsy with a larger gauge size or vacuum-assisted device, or surgical excision [12].

Given the uncommon nature of the lesion, there are no large randomized controlled trials regarding the management of breast tubular adenomas to date. Given the limited understanding of tubular adenoma compared with fibroadenoma, Miles et al. recommend excision of tubular adenoma to rule out synchronous malignancy, while patients who opt not to undergo an operation or are unable to undergo an operation should be closely monitored over time. Once excisional biopsy confirms the diagnosis of tubular adenoma, no further additional treatment is required [13].

Joo et al. recommends surgical excision or repeat core biopsy if surveillance imaging exhibits features of suspicious calcification or rapid growth of > 20% in 6 months. They described that tubular adenomas typically remain stable in size once they reach a maximum size averaging 3 cm after months to years [14].

Surveillance imaging at 12–18 months revealed no radiological changes for Cases A and B, and as such excisional biopsy was not performed. Cases C, D, E, and F highlight the benign surveillance outcomes for patients who underwent excisional biopsy confirming tubular adenoma. These findings align with the management recommendations by Miles and Joo, and add to the body of evidence of safe management of breast tubular adenomas.
